# *Chlamydia trachomatis* Pgp3 Antibody Population Seroprevalence before and during an Era of Widespread Opportunistic Chlamydia Screening in England (1994-2012)

**DOI:** 10.1371/journal.pone.0152810

**Published:** 2017-01-27

**Authors:** Sarah C. Woodhall, Gillian S. Wills, Patrick J. Horner, Rachel Craig, Jennifer S. Mindell, Gary Murphy, Myra O. McClure, Kate Soldan, Anthony Nardone, Anne M. Johnson

**Affiliations:** 1 National Infection Service, Public Health England, London, United Kingdom; 2 Research Department of Infection and Population Health, UCL, London, United Kingdom; 3 Jefferiss Research Trust Laboratories, Faculty of Medicine, Imperial College London, St Mary's Campus, London, United Kingdom; 4 School of Social and Community Medicine, University of Bristol, Bristol, United Kingdom; 5 NatCen Social Research, London, United Kingdom; 6 Research Department of Epidemiology and Public Health, UCL, London, United Kingdom; University of Texas Health Science Center at San Antonio, UNITED STATES

## Abstract

**Background:**

Opportunistic chlamydia screening of <25 year-olds was nationally-implemented in England in 2008 but its impact on chlamydia transmission is poorly understood. We undertook a population-based seroprevalence study to explore the impact of screening on cumulative incidence of chlamydia, as measured by *C*.*trachomatis*-specific antibody.

**Methods:**

Anonymised sera from participants in the nationally-representative Health Surveys for England (HSE) were tested for *C*.*trachomatis* antibodies using two novel Pgp3 enzyme-linked immunosorbent assays (ELISAs) as a marker of past infection. Determinants of being seropositive were explored using logistic regression among 16–44 year-old women and men in 2010 and 2012 (years when sexual behaviour questions were included in the survey) (n = 1,402 women; 1,119 men). Seroprevalence trends among 16–24 year-old women (n = 3,361) were investigated over ten time points from 1994–2012.

**Results:**

In HSE2010/2012, Pgp3 seroprevalence among 16–44 year-olds was 24.4% (95%CI 22.0–27.1) in women and 13.9% (11.8–16.2) in men. Seroprevalence increased with age (up to 33.5% [27.5–40.2] in 30–34 year-old women, 18.7% [13.4–25.6] in 35–39 year-old men); years since first sex; number of lifetime sexual partners; and younger age at first sex. 76.7% of seropositive 16–24 year-olds had never been diagnosed with chlamydia. Among 16–24 year-old women, a non-significant decline in seroprevalence was observed from 2008–2012 (prevalence ratio per year: 0.94 [0.84–1.05]).

**Conclusion:**

Our application of Pgp3 ELISAs demonstrates a high lifetime risk of chlamydia infection among women and a large proportion of undiagnosed infections. A decrease in age-specific cumulative incidence following national implementation of opportunistic chlamydia screening has not yet been demonstrated. We propose these assays be used to assess impact of chlamydia control programmes.

## Background

Genital infection with *Chlamydia trachomatis* (‘chlamydia’) is the most commonly-diagnosed sexually transmitted infection (STI) in the UK,[1] and an important cause of pelvic inflammatory disease, ectopic pregnancy and tubal factor infertility in women[2–5]. Many chlamydia infections are asymptomatic[6;7] so can go undiagnosed. In England, the National Chlamydia Screening Programme (NCSP) recommends opportunistic screening for chlamydia annually and on change of sexual partner for sexually-active under-25 year-olds with the aim of detecting and treating asymptomatic infections to reduce transmission and complications[8]. The national implementation and scale-up of the NCSP in 2008 drove a large increase in chlamydia screening, such that 2.3 million tests were reported in 2010 among 15- to 24-year-olds, equivalent to 44% of women and 24% of men in this age group[9].

Chlamydia screening at the levels now seen in England is expected to reduce the incidence and prevalence of chlamydia infection among the general population[10]. However, evaluating the real-world impact of chlamydia screening presents a considerable challenge, in part due to the absence of a robust outcome measure. Routine data on chlamydia diagnoses do not provide good evidence of chlamydia incidence or prevalence in the general population as infections are often asymptomatic and numbers of diagnoses depend on the proportion and risk characteristics of the population tested[2;11]. Population-based estimates of the prevalence of current chlamydia infections (i.e. using nucleic acid amplification tests, NAATs) are resource-intensive and hard to achieve[12].

Given these challenges, studies that measure the prevalence of antibodies in serum have been proposed as a means of evaluating the impact of chlamydia control programmes[13]. Serological testing for *C*.*trachomatis*-specific antibodies has previously been problematic as available assays had poor sensitivity and specificity and were often cross-reactive with other chlamydiae species[14;15]. In recent years, tests with better sensitivity and specificity have become available and offer new potential for the evaluation of chlamydia control programmes[16;17]Antibodies to the highly immunogenic and specific *C*.*trachomatis* Pgp3 protein[18;19] persist following infection, thus providing a marker of past infection. This in turn allows estimation of age-specific cumulative incidence, which should be informative for evaluating the impact of chlamydia screening against its aims of reducing transmission[17;20].

We used data and stored sera from nationally-representative household surveys from 1994 to 2012 to explore sociodemographic and behavioural factors associated with serological evidence of a previous infection and to evaluate the impact of widespread opportunistic chlamydia screening on age-specific cumulative incidence of chlamydia in England up to 2012.

## Methods

### Participants

The Health Survey for England (HSE) is a nationally-representative survey carried out annually since 1991. Participants are invited to provide a blood sample for laboratory analyses and storage for future research. Details of HSE methodology are reported elsewhere[21;22]. In summary, each year’s survey used a stratified probability sampling design. Households were selected annually from a national postcode list. Residents aged ≥16 years (up to 10 per household) were eligible for interview (children were eligible but are not part of this study). Health and sociodemographic information was collected using face-to-face interviews, self-completed questionnaire booklets and a nurse visit. In 2010, HSE for the first time included questions on sexual behaviours and chlamydia diagnosis history, collected using the self-completed booklet. These were repeated in 2012.

Stored sera from HSE participants who had provided a specimen with informed consent for future use were obtained from a) 16- to 44-year-old male and female HSE 2010 and HSE 2012 participants (hereafter HSE2010/2012) to explore factors associated with being Pgp3 seropositive and b) female participants aged 16 to 24 who took part in HSE years when stored sera were available (1994–1996, 2001–02, and each year 2008–2012), to examine seroprevalence trends in the NCSP target age group. Time trends in men were not investigated due to lower assay sensitivity[16;17].

### Laboratory testing

Our testing strategy used two ‘in-house’ enzyme-linked immunosorbent assays (ELISAs) based on the *C*.*trachomatis*-specific antigen Pgp3. Pgp3 is transcribed from the highly conserved *C*.*trachomatis* cryptic plasmid, which has not been found in human *C*. *pneumoniae* isolates[23] and has been found to be highly immunogenic in its native, trimeric form[18;19]. Pgp3 is thus a specific (i.e. not subject to cross-reaction) and potentially highly sensitive marker of previous infection. Pgp3 is associated with the bacterial outer membrane and secreted into the cell cytosol[24] and is a virulence factor supporting *C*.*trachomatis* infection[25].

All specimens were first tested using an indirect Pgp3 ELISA, the performance characteristics of which have been previously described.[16] Briefly, sensitivity to detect a previous known infection was assessed among women and men with a clinical diagnosis of chlamydia (range 0 to >1500 days between diagnosis and blood sample) and was found to be 73.8% (66.5–79.9) in women and 44.2% (37.3–51.3) in men[16;20]. Specificity was estimated using microimmunofluorescence (MIF)-negative paediatric sera to reduce likelihood of a previous sexually-acquired *C*. *trachomatis* infection, and was found to be 97.6% (95%CI 96.2%-98.6%)[16]. The second assay used in our testing strategy was a double-antigen sandwich ELISA (hereafter ‘double-antigen ELISA’). As reported by Horner *et al*, the double-antigen ELISA has demonstrated equivalent specificity (97.8%, 95% CI 96.5–99.1), but higher sensitivity (82.9%, 77.0–88.8 in women; 54.4%, 47.2–61.6 in men) than the Pgp3 indirect ELISA when evaluated against the same clinical samples[17]. As also reported by Horner *et al* the two assays used in our detection strategy have been found to be more sensitive to detect a previous known infection than both the MIF, which detects *C*.*trachomatis* antibodies to the elementary body form of the bacteria[26], and commercial assays that target the Major Outer Membrane Protein (MOMP; Anilabsystems, SeroCT, Medac) [17]. In women, sensitivities of the Pgp3 double-antigen and indirect ELISAs were found to be 23% and 12% higher respectively than the best-performing commercial assay (Anilabsystems, sensitivity 59.5%)[17]. Among men, the sensitivity was 14% and 7% greater than for the Anilabsystems assay (sensitivity 40.2%). Sensitivity among men was lower than that seen in women for both Pgp3 ELISAs and the MOMP and MIF assays[17].

Although the double-antigen ELISA has demonstrated higher sensitivity than the indirect ELISA, the double-antigen ELISA requires around a 25-fold higher volume of sera. A separate comparison of results from sera tested on both assays showed that the indirect ELISA has good agreement with the double-antigen ELISA at low (<0.1) and high (>1.0) absorbance values.[17] We therefore used the indirect ELISA for initial screening, with subsequent testing of sera with absorbance values between 0.1 and 1.0 using the double-antigen ELISA to resolve ‘equivocal’ specimens (Table 1).

**Table 1:** Pgp3 antibody test result according to testing strategy.

		Double-antigen sandwich ELISA result	
		Negative	Positive
**Indirect ELISA result (Absorbance range)**	**Negative (<0.1)**	Pgp3 negative	N/A: Not re-tested
	Negative (0.1–0.4730)*	Pgp3 negative	Pgp3 positive
	**Positive (0.4731–1.0)**	Pgp3 positive	Pgp3 positive
	**Positive (>1.0)**	Pgp3 positive	N/A: Not re-tested

*An absorbance (450-620nm) value of 0.473 is the cutoff for the indirect assay.

### Statistical analyses

Data were analysed in Stata 12.1 accounting for weighting, clustering and stratification. Weights were applied in line with HSE analysis guidelines and included weights to correct for uneven probability of selection and, from HSE2003 onwards, non-response weights to ensure the sample is representative with regard to age, sex and region and to adjust for differential participation in blood specimen collection by sociodemographic and general health variables[27]. The sample size was determined by the number of residual sera available from the surveys with consent for future testing.

The seroprevalence of Pgp3 antibodies (hereafter termed ‘Pgp3 seroprevalence’) among HSE2010/2012 participants was estimated by sex, number of lifetime sexual partners and previous chlamydia diagnosis, regardless of reported sexual activity. Associations between having Pgp3 antibodies detected in serum (‘Pgp3 seropositive’) and sociodemographic and behavioural factors were then examined among participants reporting at least one sexual partner by the time of the interview (hereafter termed ‘sexually-experienced’) using univariable and multivariable logistic regression. Associations with deprivation were explored using a residence-based measure, the quintile of Index of Multiple Deprivation (IMD) for the lower-layer super output area (LSOA) of residence (a geographical area of around 1,500 people[28]). Sexual behaviour variables reflecting lifetime (years since first heterosexual sex, number of lifetime sexual partners) and more recent exposures (number of sexual partners in the last year, condom used at last sex) were included. Number of lifetime sexual partners was *a priori* considered the main mechanism of exposure to infection. Odds ratios (ORs) adjusted for number of lifetime sexual partners were therefore calculated to reduce confounding of the association between other predictor variables and being Pgp3 seropositive.

Pgp3 seroprevalence was estimated among 16- to 24-year-old women for years with available sera from 1994 to 2012. The trend in Pgp3 seroprevalence was estimated from 2008 (the first year when the NCSP was nationally-available) to 2012 using a generalised linear model with year entered as a continuous variable. Seroprevalence was explored by birth cohort among women aged 16 to 24, with cohorts grouped to reflect their relative exposure to widespread chlamydia screening. Women who were ≤16 years in 2008 (the first year of national implementation of the NCSP) were defined as having high screening exposure; those aged 17 to 24 in 2008 were defined as having partial screening exposure and those aged >24 years in 2008 were defined as having ‘limited’ screening exposure (see S1 File).

### Ethical approval

The Health Survey for England is approved by an NHS research ethics committee each year. This use of stored sera was approved by Yorkshire and Humber-South Yorkshire Research Ethics Committee (ref:13/YH/0304). Participants included in this study all provided written informed consent to participate in the survey, to provide blood samples, and to have these stored for future anonymous testing as part of ethically approved research. Minors (those aged 16–17 years) provided their own informed consent; consent was not sought from parents or guardians.

## Results

Of 6,882 eligible participants, samples for 1,264 were unavailable due to a missing sample or insufficient residual volume. Overall, 1,111/5,618 eligible participants with a valid antibody test result were Pgp3 seropositive (see flow chart, S1 Fig).

### Pgp3 seroprevalence in HSE2010/12

Overall Pgp3 seroprevalence among 16- to 44-year-olds in HSE2010/12 was 24.4% (95% CI 22.0–27.1) in women and 13.9% (11.8–16.2) in men, thus indicating the proportion of the population who had at least one antibody-inducing infection by the time of participating in the survey. The lower seroprevalence in men may reflect the low sensitivity of the assays to detect a previous known infection[16;17]. Among individuals who reported a previous chlamydia diagnosis, 64.7% (51.9–75.6) of women and 43.9% (26.5–63.0) of men were Pgp3 seropositive.

**Fig 1:**
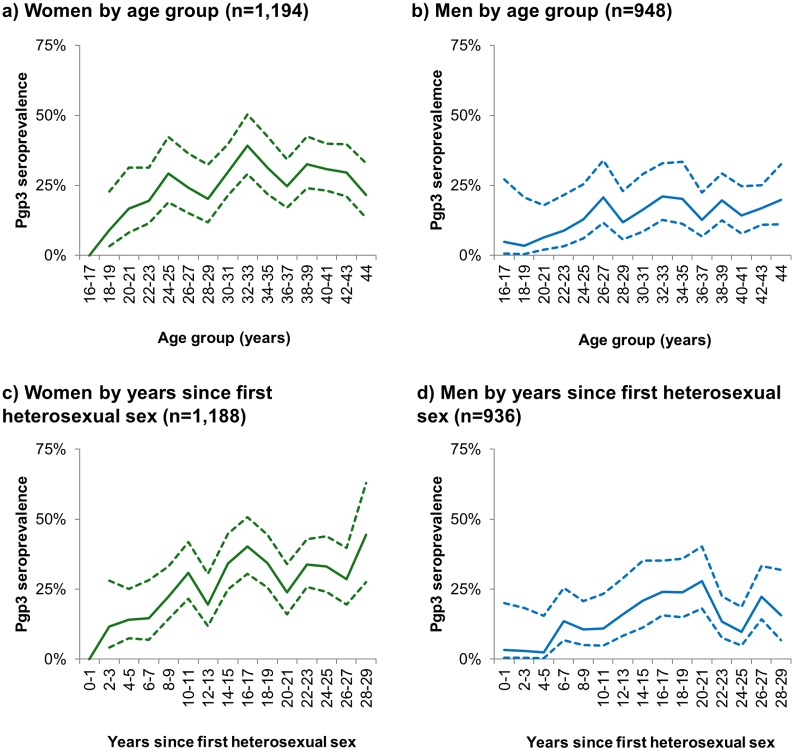
Pgp3 seroprevalence by age group (1a & 1b) and by years since first heterosexual sex (1c & 1d) (sexually-experienced 16- to 44-year-olds, HSE2010 & HSE2012). Solid lines show point estimates; dashed lines show 95% confidence intervals. N shows unweighted denominators. 95% confidence intervals are not shown in Fig 1a for 16–17 year-olds or in Fig 1b for those within 0–1 years of first heterosexual sex as no individuals in this group were Pgp3 seropositive.

**Fig 2:**
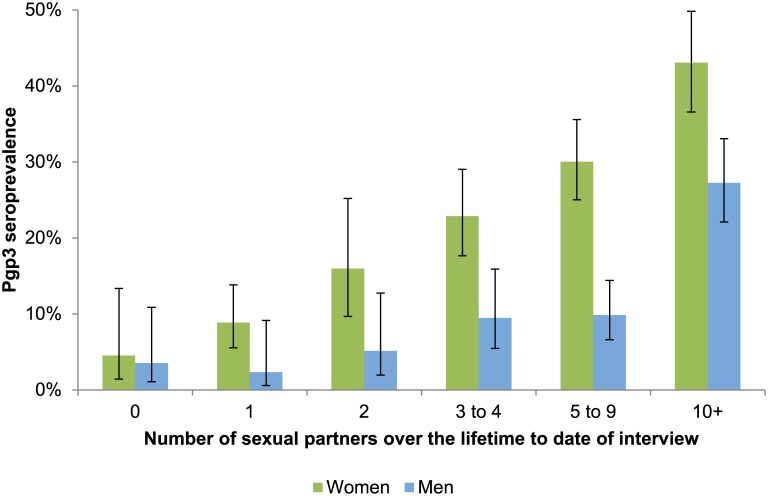
Pgp3 seroprevalence by reported numbers of lifetime sexual partners (among 16- to 44-year olds, HSE2010 & HSE2012).

Among sexually-experienced participants, Pgp3 seroprevalence increased with age and years since first sex (Fig 1; Tables 2 and 3), with peak seroprevalence in women aged 30 to 34 (33.5%) and men aged 35 to 39 (18.7%). In women, those reporting ≥10 lifetime sexual partners had almost four-fold higher odds of being Pgp3 seropositive than those with 1–4 partners (OR 3.84, 2.68–5.51). Being Pgp3 seropositive was also significantly associated with living in more deprived areas, younger age at first sex, non-condom use in the last year, and reporting a previous chlamydia diagnosis (Table 2, Fig 2). All variables associated with being Pgp3 seropositive in univariable analyses remained statistically significant after adjusting for number of lifetime sexual partners.

**Table 2:** Percentage Pgp3 seropositive by sociodemographic and sexual behavioural variables among sexually-experienced 16 to 44 year old women (HSE2010 & HSE2012).

	Pgp3 positive		Unadjusted OR		p	Adjusted OR^a^ (95%CI)		p	Denominator (W,UW)^b^
	%	95% CI	OR	95% CI		AOR	95%CI		
**Overall**	25.8	(23.1–28.6)							1423, 1194
**Age group**									
16–19	6.3	(2.3–16.4)	1.00	Ref	0.002	1.00	Ref	0.009	114, 55
20–24	21.4	(15.3–29.2)	4.05	(1.29–12.78)		3.70	(1.17–11.71)		255, 139
25–29	23.3	(17.1–30.8)	4.50	(1.44–14.07)		3.92	(1.26–12.16)		229, 166
30–34	33.5	(27.5–40.2)	7.50	(2.50–22.54)		6.34	(2.11–19.04)		284, 224
35–39	29.5	(24.2–35.6)	6.23	(2.08–18.65)		5.07	(1.71–15.06)		240, 278
40–44	28.5	(23.2–34.3)	5.91	(1.99–17.60)		5.47	(1.84–16.26)		302, 332
**IMD quintile of LSOA of residence**^c^									
Least deprived	22.7	(17.1–29.5)	1.00	Ref	0.033	1.00	Ref	0.037	256, 238
2	25.8	(20.3–32.2)	1.18	(0.74–1.89)		1.08	(0.67–1.73)		292, 248
3	19.6	(15.1–25.2)	0.83	(0.51–1.35)		0.83	(0.50–1.38)		309, 256
4	32.1	(26.1–38.8)	1.61	(1.02–2.53)		1.61	(1.00–2.61)		290, 230
Most deprived	28.9	(22.7–36.0)	1.38	(0.86–2.24)		1.46	(0.87–2.43)		276, 222
**Age at first heterosexual sex**									
16+ at first intercourse	22.5	(19.6–25.7)	1.00	Ref	0.000	1.00	Ref	0.004	1080, 911
<16 at first intercourse	39.6	(33.0–46.6)	2.26	(1.61–3.16)		1.68	(1.18–2.39)		304, 246
**Years since first heterosexual sex**									
0 to 4	8.1	(3.8–16.7)	1.00	Ref	<0.001	1.00	Ref	<0.001	184, 90
5 to 9	18.5	(13.1–25.4)	2.56	(1.01–6.49)		2.05	(0.80–5.20)		289, 174
10 to 14	25.9	(20.1–32.8)	3.95	(1.61–9.69)		2.79	(1.14–6.79)		256, 199
15 to 19	38.2	(32.0–44.7)	6.96	(2.98–16.26)		5.40	(2.31–12.62)		271, 264
20+	31.1	(26.5–36.0)	5.08	(2.19–11.77)		3.61	(1.55–8.37)		415, 461
**Number of partners of the opposite sex in last year**									
0	19.2	(11.7–30.0)	1.00	Ref	0.370	1.00	Ref	0.028	91, 81
1	26.8	(23.8–30.0)	1.53	(0.84–2.79)		1.58	(0.80–3.12)		1159, 993
2+	25.7	(17.9–35.4)	1.45	(0.69–3.06)		0.89	(0.41–1.94)		149, 103
**Number of lifetime sexual partners**^d^									
1 to 4	16.5	(13.2–20.3)	1.00	Ref	<0.001				
5 to 9	30.0	(25.0–35.6)	2.18	(1.51–3.14)					
10+	43.1	(36.6–49.8)	3.84	(2.68–5.51)					
**Was a condom used on any occasions in last 4 weeks?**									
Yes, used on every occasion	15.8	(11.4–21.6)	1.00	Ref	0.001	1.00	Ref	0.001	262, 207
Yes, used on some occasions	19.4	(12.7–28.5)	1.28	(0.68–2.41)		1.09	(0.57–2.09)		133, 95
No, not used in last 4 weeks	30.1	(26.4–34.1)	2.30	(1.49–3.53)		2.17	(1.41–3.35)		735, 630
Not had vaginal or anal sex in last 4 weeks	26.8	(20.5–34.3)	1.95	(1.15–3.31)		1.79	(1.03–3.11)		239, 209
**Ever been told by a doctor that you have chlamydia**									
No	23.6	(20.9–26.7)	1.00	Ref	<0.001	1.00	Ref	<0.001	1145, 960
Yes	65.5	(52.7–76.3)	6.12	(3.57–10.51)		5.08	(2.79–9.23)		76, 68

^a^Adjusted for lifetime sexual partners only.

^b^Analyses were conducted on those with non-missing data on the variable(s) of interest. Denominator totals vary due to item-missingness. W: Weighted; UW: Unweighted.

^c^IMD: index of multiple deprivation; LSOA: Lower super output area

^d^Includes partners of both the opposite and of the same sex.

**Table 3:** Percentage Pgp3 seropositive by sociodemographic and sexual behavioural variables among sexually-experienced 16 to 44 year old men (HSE2010 & HSE2012).

	Pgp3 positive		Unadjusted OR		p	Adjusted OR^a^ (95%CI)		p	Denominator (W,UW)^b^
	%	95% CI	OR	95% CI		AOR	95% CI		
**Overall**	14.6	(12.3–17.1)							1424, 948
**Age group**									
16–19	4.1	(1.0–15.0)	1.00	-	0.113	1.00	-	0.385	142, 66
20–24	10.1	(5.8–17.0)	2.66	(0.57–12.40)		1.74	(0.35–8.61)		220, 114
25–29	14.3	(9.2–21.5)	3.94	(0.88–17.68)		2.32	(0.48–11.20)		255, 139
30–34	18.1	(12.6–25.3)	5.22	(1.19–23.00)		3.21	(0.70–14.76)		251, 165
35–39	18.7	(13.4–25.6)	5.45	(1.24–23.96)		3.22	(0.70–14.76)		242, 214
40–44	16.7	(12.4–22.1)	4.74	(1.09–20.65)		2.74	(0.60–12.62)		314, 250
**IMD quintile of LSOA of residence**^c^									
Least deprived	13.7	(8.9–20.5)	1.00	Ref	0.680	1.00	Ref	0.795	241, 184
2	12.4	(8.5–17.8)	0.90	(0.47–1.72)		0.98	(0.50–1.92)		283, 195
3	14.8	(10.0–21.3)	1.10	(0.56–2.14)		1.31	(0.65–2.61)		304, 199
4	13.8	(9.5–19.6)	1.01	(0.54–1.88)		0.90	(0.44–1.82)		285, 179
Most deprived	17.7	(12.7–24.0)	1.36	(0.73–2.52)		1.22	(0.61–2.45)		310, 191
**Age at first heterosexual sex**									
16+ at first intercourse	12.0	(9.6–15.0)	1.00	Ref	0.002	1.00	Ref	0.233	980, 658
<16 at first intercourse	22.6	(17.5–28.8)	2.14	(1.41–3.24)		1.50	(0.94–02.38)		357, 231
**Years since first heterosexual sex**									
0 to 4	3.5	(1.1–10.5)	1.00	Ref	0.003	1.00	-	0.077	230, 109
5 to 9	10.1	(6.2–16.2)	3.11	(0.86–11.18)		1.78	(0.47–6.82)		283, 154
10 to 14	16.3	(10.7–24.0)	5.36	(1.51–19.03)		3.12	(0.85–11.42)		236, 148
15 to 19	22.3	(16.2–29.8)	7.91	(2.31–27.02)		4.10	(1.15–14.66)		249, 186
20+	18.0	(14.1–22.6)	6.03	(1.84–19.76)		3.04	(0.90–10.33)		409, 339
**Number of partners of the opposite sex in last year**									
0	10.4	(3.8–25.5)	1.00	Ref	0.741	1.00	-	0.687	100, 59
1	14.5	(12.1–17.4)	1.46	(0.49–4.35)		1.52	(0.43–5.38)		1059, 745
2+	15.6	(10.2–23.1)	1.59	(0.49–5.16)		1.28	(0.32–5.06)		215, 115
**Total number of lifetime sexual partners**^d^									
1 to 4	5.9	(3.7–9.2)	1.00	Ref	<0.001				575, 361
5 to 9	9.9	(6.6–14.4)	1.74	(0.90–3.33)					322, 223
10+	27.2	(22.1–33.1)	5.95	(3.41–10.35)					446, 306
**Was a condom used on any occasions in last 4 weeks?**									
Yes, used on every occasion	10.8	(7.0–16.3)	1.00	Ref	0.046	1.00	-	0.272	285, 181
Yes, used on some occasions	13.0	(7.6–21.2)	1.23	(0.58–2.61)		1.24	(0.54–2.82)		173, 98
No, not used in last 4 weeks	17.6	(14.3–21.4)	1.76	(1.04–3.00)		1.51	(0.83–2.72)		657, 479
Not had vaginal or anal sex in last 4 weeks	9.7	(5.7–15.8)	0.88	(0.43–1.81)		0.86	(0.39–1.90)		246, 151
**Ever been told by a doctor that you have chlamydia**									
No	12.8	(10.6–15.4)	1.00	Ref	<0.001	1.00	Ref	0.003	1145, 776
Yes	44.6	(26.9–63.8)	5.47	(2.47–12.15)		3.55	(1.53–8.25)		62, 35

Percentage Pgp3 seropositive in men should be interpreted with caution due to the relatively low sensitivity of the assays used to detect a known previous infection in men (see Methods section).

^a^Adjusted for lifetime sexual partners only.

^b^Analyses were conducted on those with non-missing data on the variable(s) of interest. Denominator totals vary due to item-missingness. W: Weighted; UW: Unweighted.

^c^IMD: index of multiple deprivation; LSOA: Lower super output area

^d^Includes partners of both the opposite and of the same sex.

In sexually-experienced men, similar factors were associated with being seropositive as seen in women in univariable analyses although age group and deprivation of residence were not statistically significant (Table 3, Fig 2). Men reporting ≥10 lifetime sexual partners had almost six-fold higher odds of being Pgp3 seropositive than those with 1–4 partners (OR 5.95, 3.41–10.35). After adjusting for number of lifetime sexual partners, only reporting a previous diagnosis of chlamydia remained a significant predictor of being seropositive in men (AOR 3.55, 1.53–8.25).

Among Pgp3 seropositive individuals, 84.7% (80.3–88.3) of 16- to 44-year-olds did not report a previous chlamydia diagnosis. This proportion varied by age group, whereby 76.7% of 16- to 24-year-olds, 79.0% of 25- to 34-year-olds and 92.9% of 35- to 44-year-olds did not report a previous diagnosis. Among Pgp3 seropositive 16- to 24-year-olds, 36.1% (23.1–51.5) had never been tested for chlamydia.

### Pgp3 seroprevalence over time among 16- to 24-year-old women

**Fig 3:**
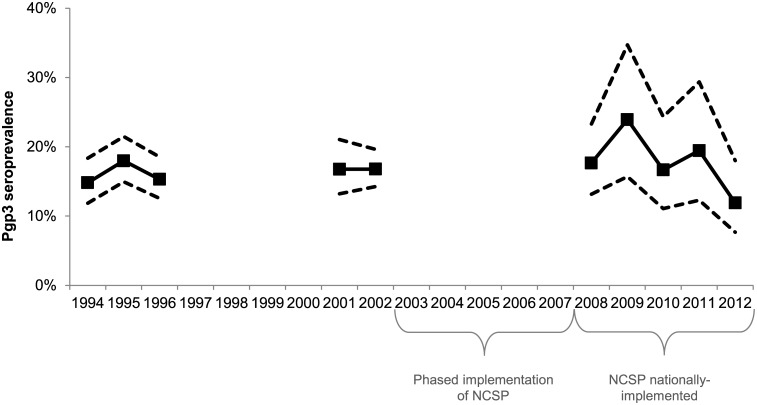
Pgp3 seroprevalence by year (16- to 24-year-old women, HSE 1994 to 2012). Solid lines show point estimates; dashed lines show 95% confidence intervals. Unweighted denominators: 1994–1996, n = 1,555; 2001–2002, n = 1097; 2008–2012, n = 709.

Fig 3 shows Pgp3 seroprevalence among 16- to 24-year-old women across the time points included between 1994 and 2012. There was no significant difference in Pgp3 seroprevalence among these women between the first (1994–1996) and second (2001–2002) time-periods sampled. Between 2008 and 2012, a non-significant decline was observed (prevalence ratio per year: 0.94, 95%CI 0.84–1.05; p = 0.26). After stratifying by age group, there was no notable trend among 16- to 19-year-olds (prevalence ratio: 0.96, 0.74–1.24; p = 0.76) and a non-significant decline in Pgp3 seroprevalence among 20- to 24-year-olds (prevalence ratio: 0.92, 0.83–1.04; p = 0.18).

**Fig 4:**
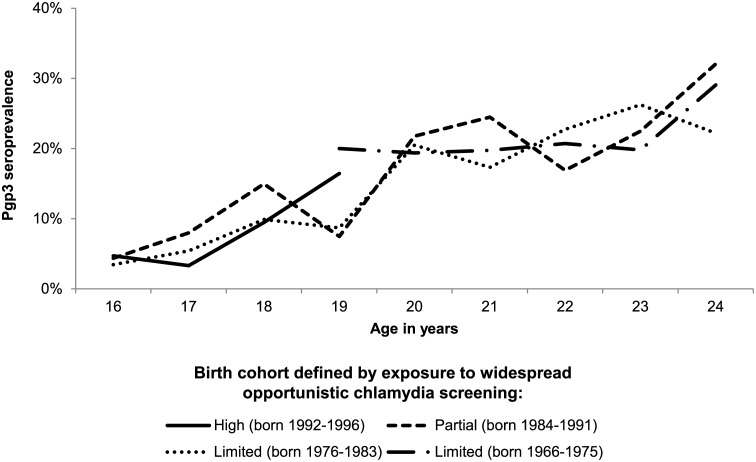
Pgp3 seroprevalence by birth cohort and year of age (16- to 24-year-old women, HSE 1994 to 2012). Unweighted denominators: High screening exposure (born 1992–1996), n = 185; Partial screening exposure (born 1984–1991), n = 853; Limited screening exposure (born 1976–1983), n = 1349; Limited screening exposure (born 1966–1975), n = 929. See online appendix (S1 File) for details of denominators by year of age.

Fig 4 shows Pgp3 seroprevalence by year of age and birth cohort defined by exposure to chlamydia screening. Although only partial data were available on those with high exposure to screening, there was no indication of a difference in the age-specific seroprevalence by birth cohort, with similar age curves seen in each group.

## Discussion

In 2010/2012, one quarter of women aged 16 to 44 and one in three of those aged 30 to 34 had evidence of a previous antibody-inducing chlamydia infection. Being Pgp3 seropositive was strongly associated with increasing age and numbers of lifetime sexual partners. Three quarters of those under-25 with evidence of previous infection did not report a previous chlamydia diagnosis, suggesting a high level of undiagnosed infections. There was no significant trend in age-specific Pgp3 seroprevalence over time or between birth cohorts exposed to different levels of opportunistic chlamydia screening.

A major strength of our study is our use of sera from a series of nationally-representative samples, which incorporated sociodemographic and (in HSE2010/2012) sexual behavioural data. We used assays that have demonstrated higher sensitivity and specificity than commercially-available tests[16;17]. There were some limitations. Our findings may be affected by who agreed to participate in the survey or provide a blood sample, although non-response weights were applied to account for non-participation and HSE2010/2012 participants who contributed to this study were comparable to the overall HSE population on a range of sociodemographic and behavioural variables (S1 Table). Another limitation is that behavioural data were self-reported. Sensitive items were collected using the self-completion booklet to minimise social desirability bias. However, as data were self-reported and as household members could be present during booklet completion[21], underreporting remains possible[29].

Given the sensitivity of the double-antigen ELISA (82.9% in women; 54.4% in men)[17], the proportion of the population ever infected with chlamydia would be even higher than we estimated. Pgp3 antibodies were detected among a small proportion of people who did not report having ever had sexual intercourse (4.5% of women; 3.5% of men; Fig 2). This may be due to under-reporting of sexual experience or to false positives that would be expected from low prevalence populations. None of the sexually-experienced 16- to 17-year-old women were seropositive for Pgp3. This finding was unanticipated, as we would expect some evidence of previous infection in this age group. In the third National Survey of Sexual Attitudes and Lifestyles (Natsal-3; a nationally-representative survey of adults living in Britain, conducted in 2010–12), twenty-nine of percent 16- to 24-year-olds reported having first had sex before age 16[30]. Furthermore, chlamydia diagnoses have been reported in national surveillance data among those aged under 16 years[31]. Although we would, therefore, expect a proportion of 16- to 17- year-old women to have been infected with chlamydia, our analysis was limited by the sample size available (n = 18); a larger sample size might have detected evidence of previous infection among the youngest age group. Seroprevalence patterns in men should be interpreted with caution given the relatively low sensitivity of chlamydia antibody to detect previous infection in men[16;17]. This lower sensitivity, which has also been noted with other assays[17], may indicate that men are less likely to mount a persistent antibody response to *C*.*trachomatis* infection.

The percent Pgp3 seropositive among those reporting a previous diagnosis of chlamydia was lower than the estimated sensitivity of the assays used (e.g. 64.7% (51.9–75.6) in women versus 82.9% (77.0–88.8) sensitivity of the double-antigen ELISA)[15]. This is similar to findings from the New Zealand Dunedin Multidisciplinary Health and Development Study studied by Horner et al., where 74.6% (61.6–85.0) of 448 women aged 38 years with previous history of chlamydia infection had detectable Pgp3 antibody using the double-antigen ELISA [15]. This may be due to reporting error in chlamydia history as people can forget or misunderstand the STI they had, or alternatively reflect differences in the age of participants in our study compared with the clinical population used for assay validation, thus indicating some waning of detectable antibodies over time. In their 2013 study, Horner et al demonstrated that Pgp3 seropositivity measured using the indirect ELISA alone declines with time since infection, with the most apparent declines seen in the first six months after diagnosis[20]. However, Horner et al’s more recent study of female participants in a cohort study in New Zealand, which used the double-antigen ELISA, found that over 95% of women who were seropositive at age 26 were still seropositive at age 38, suggesting persistence up to at least 12 years following infection[17]. This suggests our study, which incorporated the double-antigen assay into the detection strategy, will be less subject to declines in detectable antibodies over time than if the indirect ELISA alone had been used. Nevertheless, given the potential impact of assay sensitivity on interpreting trends in seroprevalence, the duration of antibody response needs further clarification. Work is therefore ongoing to better characterise Pgp3 antibody response using the two Pgp3 ELISAs used in relation to time since infection and number of previous infections[32].

Pgp3 seroprevalence plateaued in women from around the age of 30. This may reflect a reduction in acquisition of infection in older women, which would be consistent with the prevalence of *current* chlamydia infection peaking in those under-25 [33]. This may also reflect differences in cumulative exposure to chlamydia infection by birth cohort, given the decline in STI rates observed in the mid-1980s to the mid-1990s and the subsequent rise[34]. A previous population-based study in the Netherlands using the Medac CT IgG ELISA found a less marked relationship with age than was seen in our study, as well as a lower overall seroprevalence (9.8% in women; 5.7% in men aged 15 to 39[35]). These differences may be due to the difference in assay sensitivity to detect a previous known infection (Medac: 46.2% versus double-antigen ELISA: 82.9%)[17].

We found no significant change in age-specific Pgp3 seroprevalence between 2008 and 2012 and no difference in seroprevalence between birth cohorts exposed to different levels of opportunistic chlamydia screening. This is perhaps surprising given the increase in chlamydia control efforts over the last decade. There are a number of possible explanations for this lack of a decline. First, high levels of chlamydia screening may not have been in place long enough to effect a change in age-specific cumulative incidence, given that the NCSP was only nationally-implemented in 2008. Secondly, our analysis of trends over time was limited by the number of samples available; a larger sample might have had power to detect a change. However it should be noted that the observed (non-significant) decrease in seroprevalence between 2008 and 2012 was especially influenced by seroprevalence in 2012 among 20- to 24-year-olds (S2 Fig). In contrast to our study, Horner *et a*l found a significant decline in Pgp3 seroprevalence from 2007 to 2010 among 17- to 24-year-old women[36]. The difference in findings may be due to sources of sera (Horner *et al* used residual sera submitted to laboratories for routine investigations rather than a probability sample) or the different time-periods investigated. However an alternative explanation is also suggested by our analysis. We found that at least a third of women will be infected with chlamydia at some point over their lifetime and that the majority of seropositive individuals in HSE2010/2012 had never been diagnosed. We propose that this combination of high lifetime cumulative incidence and large undiagnosed fraction offers a partial explanation for the absence of a decline in seroprevalence among 16- to 24-year-old women up to 2012.

Our findings suggest that screening activity up to 2010/2012 was leaving the majority of infections in under-25 year-olds undiagnosed. Nevertheless, 64% of Pgp3 seropositive 16- to 24-year olds had been tested for chlamydia at some time before the interview even if they had never been diagnosed. This suggests that the timing and frequency of testing in relation to infection warrants attention. Current guidance recommends chlamydia testing on change of sexual partner[8], but the extent to which this is done in practice is unclear. We also found that Pgp3 seropositivity was higher among women who reported younger age at first sex and seroprevalence increased sharply from age 16 to 25. Consistent with previous studies[17;36;37], this emphasises the importance of adolescence and young adulthood as periods when chlamydia prevention and screening activities are needed to prevent development of adverse consequences.

Our findings can also be used to inform future research in the field of chlamydia epidemiology and control. First, our estimates of age-specific cumulative incidence and proportion of infections diagnosed should be considered as potential data sources to parameterise and validate mathematical models of chlamydia screening and better understand *C*. *trachomatis* infection transmission dynamics. Secondly, our population-based estimates of Pgp3 seroprevalence by age and numbers of sexual partners provide important information for designing, implementing and evaluating future chlamydia vaccination programmes. Although not yet available, there have been several advancements towards a chlamydia vaccine in recent years[38;39]. In the event of a safe and efficacious vaccine becoming available, having an understanding of exposure to chlamydia in the general population and a means of measuring burden of chlamydia infection will be essential. Our study provides information to inform the development of such programmes and demonstrates the utility of antibody seroprevalence as a biological outcome measure for evaluating chlamydia control and prevention efforts, including future vaccination programmes.

In conclusion, we have demonstrated a novel method of assessing population levels of prior chlamydia infection using a sensitive and specific testing strategy. A significant decrease in the proportion of the population aged 16 to 24 ever infected with chlamydia following the national implementation of opportunistic chlamydia screening has not yet been demonstrated. This may be due to insufficient time since implementation of widespread screening, limited statistical power, or to the extent of infection within the general population and the high proportion of infections that, up to 2012, appeared to have gone undiagnosed. We propose that use of the Pgp3 indirect and double-antigen ELISAs in representative population samples of women provides a valid and generalisable approach to evaluating the impact of public health interventions to control chlamydia transmission.

## Supporting Information

S1 File Categorisation by birth cohort. (DOCX)

S1 Fig Flow chart showing selection of stored sera from Health Survey for England (HSE) participants and inclusion in analyses. (DOCX)

S2 Fig Pgp3 seroprevalence by year and age group (16- to 24-year-old women, HSE 2008 to 2012). Solid lines show point estimates; dashed lines show 95% confidence intervals. Unweighted denominators: 16–19 year-olds, n = 284; 20–24 year-olds, n = 425.
(TIF)

S1 Table Comparison of reported demographic and behavioural variables between HSE participants and the study population by sex and age group (16–44 year-olds, HSE2010 & HSE2012). (DOCX)

## References

[1] Public Health England. Table 8: Number and rates of selected STI diagnoses in the UK, 2008–2012. 2014. 2014.

[2] Gottlieb SL, Martin DH, Xu F, Byrne GI, Brunham RC. Summary: The natural history and immunobiology of Chlamydia trachomatis genital infection and implications for Chlamydia control. J Infect Dis 2010 6 15;201 Suppl 2:S190–S204. 20524236 10.1086/652401

[3] Price MJ, Ades A, Welton NJ, Macleod J, Turner K, Simms I, et al How much tubal factor infertility is caused by Chlamydia? Estimates based on serological evidence corrected for sensitivity and specificity. Sex Transm Dis 2012 8;39(8):608–13. 10.1097/OLQ.0b013e3182572475 22801343

[4] Price MJ, Ades AE, De AD, Welton NJ, Macleod J, Soldan K, et al Risk of pelvic inflammatory disease following Chlamydia trachomatis infection: analysis of prospective studies with a multistate model. Am J Epidemiol 2013 8 1;178(3):484–92. 10.1093/aje/kws583 23813703 PMC3727337

[5] Westrom L, Joesoef R, Reynolds G, Hagdu A, Thompson SE. Pelvic inflammatory disease and fertility. A cohort study of 1,844 women with laparoscopically verified disease and 657 control women with normal laparoscopic results. Sex Transm Dis 1992 7;19(4):185–92. 1411832

[6] Low N, McCarthy A, Macleod J, Salisbury C, Campbell R, Roberts TE, et al Epidemiological, social, diagnostic and economic evaluation of population screening for genital chlamydial infection. Health Technol Assess 2007 3;11(8):iii–xii, 1 17311735 10.3310/hta11080

[7] Stamm WE. *Chlamydia trachomatis* infections of the adult In: Holmes KK, Sparling PF, Mardh P-A,., Lemon SM, Stamm WE, et al, editors. Sexually Transmitted Diseases.New York: 1999.

[8] Public Health England. National Chlamydia Screening Programme Standards (7th edition). 2014.

[9] Health Protection Agency. Genital Chlamydia trachomatis diagnoses in young adults in England, 2011. 2012.

[10] Turner KM, Adams EJ, LaMontagne DS, Emmett L, Baster K, Edmunds WJ. Modelling the effectiveness of chlamydia screening in England. Sex Transm Infect 2006 12;82(6):496–502. 10.1136/sti.2005.019067 17151036 PMC2563876

[11] MIller WC. Epidemiology of chlamydial infection: are we losing ground? Sexually Transmitted Infections 2008;84:82–6. 10.1136/sti.2007.028662 18372493

[12] Redmond SM, Alexander-Kisslig K, Woodhall SC, van den Broek IV, van BJ, Ward H, et al Genital Chlamydia prevalence in europe and non-European high income countries: systematic review and meta-analysis. PLoS One 2015;10(1):e0115753 10.1371/journal.pone.0115753 25615574 PMC4304822

[13] Johnson AM, Horner P. A new role for Chlamydia trachomatis serology? Sex Transm Infect 2008 4;84(2):79–80. 10.1136/sti.2007.028472 18256104

[14] Persson K. The role of serology, antibiotic susceptibility testing and serovar determination in genital chlamydial infections. Best Pract Res Clin Obstet Gynaecol 2002 12;16(6):801–14. 12473283 10.1053/beog.2002.0321

[15] Land JA, van Bergen JE, Morre SA, Postma MJ. Epidemiology of Chlamydia trachomatis infection in women and the cost-effectiveness of screening. Hum Reprod Update 2010 3;16(2):189–204. 10.1093/humupd/dmp035 19828674

[16] Wills GS, Horner PJ, Reynolds R, Johnson AM, Muir DA, Brown DW, et al Pgp3 antibody enzyme-linked immunosorbent assay, a sensitive and specific assay for seroepidemiological analysis of Chlamydia trachomatis infection. Clin Vaccine Immunol 2009 6;16(6):835–43. 10.1128/CVI.00021-09 19357314 PMC2691054

[17] Horner PJ, Wills GS, Righarts A, Vieira S, Kounali D, Samuel D, et al *Chlamydia tracho*matis Pgp3 antibody persists and correlates with self-reported infection and behavioural risks in a blinded cohort study. SUBMITTED: PLoS One 2015. 10.1371/journal.pone.0151497 PMC4790965 26974653

[18] Li Z, Zhong Y, Lei L, Wu Y, Wang S, Zhong G. Antibodies from women urogenitally infected with C. trachomatis predominantly recognized the plasmid protein pgp3 in a conformation-dependent manner. BMC Microbiol 2008;8:90 10.1186/1471-2180-8-90 18541036 PMC2432062

[19] Wang J, Zhang Y, Lu C, Lei L, Yu P, Zhong G. A genome-wide profiling of the humoral immune response to Chlamydia trachomatis infection reveals vaccine candidate antigens expressed in humans. J Immunol 2010 8 1;185(3):1670–80. 10.4049/jimmunol.1001240 20581152

[20] Horner PJ, Wills GS, Reynolds R, Johnson AM, Muir DA, Winston A, et al Effect of time since exposure to Chlamydia trachomatis on chlamydia antibody detection in women: a cross-sectional study. Sex Transm Infect 2013 8;89(5):398–403. 10.1136/sextrans-2011-050386 23430706

[21] Mindell J, Biddulph JP, Hirani V, Stamatakis E, Craig R, Nunn S, et al Cohort profile: the health survey for England. Int J Epidemiol 2012 12;41(6):1585–93. 10.1093/ije/dyr199 22253315

[22] Craig R, Mindell J, (eds). Health Survey for England 2012 Volume 2 Methods and documentation. Leeds: Health and Social Care Information Centre; 2013.

[23] Myers GS, Mathews SA, Eppinger M, Mitchell C, O'Brien KK, White OR, et al Evidence that human Chlamydia pneumoniae was zoonotically acquired. J Bacteriol 2009 12;191(23):7225–33. 10.1128/JB.00746-09 19749045 PMC2786552

[24] Chen D, Lei L, Lu C, Galaleldeen A, Hart PJ, Zhong G. Characterization of Pgp3, a Chlamydia trachomatis plasmid-encoded immunodominant antigen. J Bacteriol 2010 11;192(22):6017–24. 10.1128/JB.00847-10 20851898 PMC2976438

[25] Hou S, Dong X, Yang Z, Li Z, Liu Q, Zhong G. Chlamydial Plasmid-Encoded Virulence Factor Pgp3 Neutralizes the Antichlamydial Activity of Human Cathelicidin LL-37. Infect Immun 2015 12;83(12):4701–9. 10.1128/IAI.00746-15 26416907 PMC4645396

[26] Wang SP, Grayston JT. Human serology in Chlamydia trachomatis infection with microimmunofluorescence. J Infect Dis 1974 10;130(4):388–97. 4613759 10.1093/infdis/130.4.388

[27] Health & Social Care Information Centre. Health Survey for England 2008 Volume 2 Methods and documentation. 2009.

[28] Payne R.A., Abel GA. UK indices of multiple deprivation—a way to make comparisons across constiuent countries easier. Health Statistics Quarterly 2012;53.

[29] Prah P. Asking about Sex in General Health Surveys: Comparing Data Collected By the 2010 Health Survey for England with the Third Natsal Survey of Sexual Attitudes and Lifestyles. Oral presentation (3F4. at the 2014 STD Prevention Conference. Atlanta, GA, USA. Sexually Transmitted Diseases 2014;41(June, Supplement 1).

[30] Mercer CH, Tanton C, Prah P, Erens B, Sonnenberg P, Clifton S, et al Changes in sexual attitudes and lifestyles in Britain through the life course and over time: findings from the National Surveys of Sexual Attitudes and Lifestyles (Natsal). Lancet 2013 11 30;382(9907):1781–94. 10.1016/S0140-6736(13)62035-8 24286784 PMC3899021

[31] Public Health England. Sexually transmitted infections and chlamydia screening in England, 2014. Health Protection Report 2015;9(22).

[32] UK Clinical Research Network. UK Clinical Research Network: Portfolio Database. Measurement of chlamydia antibodies using residual GUM specimens (UKCRN 18521). 2015. 27-4-2015.

[33] Sonnenberg P, Clifton S, Beddows S, Field N, Soldan K, Tanton C, et al Prevalence, risk factors, and uptake of interventions for sexually transmitted infections in Britain: findings from the National Surveys of Sexual Attitudes and Lifestyles (Natsal). Lancet 2013 11 30;382(9907):1795–806. 10.1016/S0140-6736(13)61947-9 24286785 PMC3899025

[34] Nicoll A, Hughes G, Donnelly M, Livingstone S, De Angelis D, Fenton K, et al Assessing the impact of national anti-HIV sexual health campaigns: trends in the transmission of HIV and other sexually transmitted infections in England. Sex Transm Infect 2001 8;77(4):242–7. 10.1136/sti.77.4.242 11463922 PMC1744349

[35] van AF, de MM, Morre SA, van Bergen JE, van der Klis FR, Land JA, et al Chlamydia trachomatis IgG seroprevalence in the general population of the Netherlands in 1996 and in 2007: differential changes by gender and age. Sex Transm Infect 2014 8;90(5):434–40. 10.1136/sextrans-2013-051074 24583966

[36] Horner P, Soldan K, Vieira SM, Wills GS, Woodhall SC, Pebody R, et al C. trachomatis pgp3 Antibody Prevalence in Young Women in England, 1993–2010. PLoS One 2013;8(8):e72001 10.1371/journal.pone.0072001 23991024 PMC3749119

[37] Price MJ, Ades AE, De AD, Welton NJ, Macleod J, Turner K, et al Incidence of Chlamydia trachomatis infection in women in England: two methods of estimation. Epidemiol Infect 2014 3;142(3):562–76. 10.1017/S0950268813001027 23759367 PMC3915754

[38] Hafner LM, Wilson DP, Timms P. Development status and future prospects for a vaccine against *Chlamydia trachomatis* infection. Vaccine 2014;32(14). 10.1016/j.vaccine.2013.08.020 23973245

[39] Stary G, Olive A, Radovic-Moreno AF, Gondek D, Alvarez D, Basto PA, et al VACCINES. A mucosal vaccine against Chlamydia trachomatis generates two waves of protective memory T cells. Science 2015 6 19;348(6241):aaa8205 10.1126/science.aaa8205 26089520 PMC4605428

