# Supplementary material for: Chlamydia trachomatis Pgp3 Antibody Population Seroprevalence before and during an Era of Widespread Opportunistic Chlamydia Screening in England (1994-2012)
Source: PLoS One. 2017 Jan 27;12(1):e0152810. doi: 10.1371/journal.pone.0152810 (PMC5271337; doi:10.1371/journal.pone.0152810)
Supplement: S1 File — (DOCX) [file pone.0152810.s001.docx]

*Chlamydia trachomatis* Pgp3 antibody population seroprevalence before and during an era of widespread opportunistic chlamydia screening in England (1994-2012)

Supporting File 1: Categorisation by birth cohort

We explored Pgp3 seroprevalence by birth cohort among women aged 16 to 24, with birth cohorts grouped to reflect their relative exposure to widespread chlamydia screening. The median age at first sex reported among 16 to 24 year-olds in the third National Survey of Sexual Attitudes and Lifestyles (Natsal-3) was 16,^1^ and was therefore used as the proxy age of sexual debut for the purposes of categorisation by birth cohort. Women who were ≤16 years in 2008 (the first year of national implementation of the NCSP) were defined as having high exposure. Women aged 17 to 24 in 2008 were defined as having partial exposure as they would have had some of their years post-sexual debut before the NCSP was nationally-implemented but would have still been within the target age group when national implementation occurred. Women aged over 24 years in 2008 were defined as having ‘limited’ exposure as they would have been outside the target age group when the NCSP was nationally implemented.

The numbers in each group by year of age are set out in Supplementary Table 1 below.

Supplementary Table 1: Numbers of women aged 16 to 24 contributing to Pgp3 seroprevalence estimates by birth cohort and year of age

| **Exposure to widespread screening** | **Year born** | **Year turned 16** | **Number of women by year of age** | | | | | | | | | |
| --- | --- | --- | --- | --- | --- | --- | --- | --- | --- | --- | --- | --- |
|  |  |  | **16** | **17** | **18** | **19** | **20** | **21** | **22** | **23** | **24** | **16-24** |
| Limited (born 1966-1975) | 1970 | 1986 |  |  |  |  |  |  |  |  | 66 | 66 |
| 25 or older in 2008 | 1971 | 1987 |  |  |  |  |  |  |  | 61 | 81 | 142 |
|  | 1972 | 1988 |  |  |  |  |  |  | 63 | 76 | 87 | 226 |
|  | 1973 | 1989 |  |  |  |  |  | 47 | 66 | 70 |  | 183 |
|  | 1974 | 1990 |  |  |  |  | 52 | 50 | 69 |  |  | 171 |
|  | 1975 | 1991 |  |  |  | 40 | 46 | 55 |  |  |  | 141 |
| **Total** |  |  | **0** | **0** | **0** | **40** | **98** | **152** | **198** | **207** | **234** | **929** |
| Limited (born 1976-1983) | 1976 | 1992 |  |  |  | 52 | 56 |  |  |  |  | 108 |
| 25 or older in 2008 | 1977 | 1993 |  | 51 | 41 | 44 |  |  |  |  | 45 | 181 |
|  | 1978 | 1994 | 40 | 54 | 55 |  |  |  |  | 46 | 76 | 271 |
|  | 1979 | 1995 | 68 | 61 |  |  |  |  | 40 | 87 |  | 256 |
|  | 1980 | 1996 | 65 |  |  |  |  | 31 | 87 |  |  | 183 |
|  | 1981 | 1997 |  |  |  |  | 48 | 82 |  |  |  | 130 |
|  | 1982 | 1998 |  |  |  | 39 | 75 |  |  |  |  | 114 |
|  | 1983 | 1999 |  |  | 37 | 69 |  |  |  |  |  | 106 |
| **Total** |  |  | **173** | **166** | **133** | **204** | **179** | **113** | **127** | **133** | **121** | **1349** |
| Partial (born 1984-1991) | 1984 | 2000 |  | 29 | 74 |  |  |  |  |  | 36 | 139 |
| 17 to 24 in 2008 | 1985 | 2001 | 37 | 107 |  |  |  |  |  | 27 | 9 | 180 |
|  | 1986 | 2002 | 88 |  |  |  |  |  | 23 | 11 | 21 | 143 |
|  | 1987 | 2003 |  |  |  |  |  | 21 | 9 | 17 | 19 | 66 |
|  | 1988 | 2004 |  |  |  |  | 26 | 7 | 15 | 24 | 21 | 93 |
|  | 1989 | 2005 |  |  |  | 16 | 8 | 15 | 11 | 20 |  | 70 |
|  | 1990 | 2006 |  |  | 22 | 6 | 10 | 20 | 20 |  |  | 78 |
|  | 1991 | 2007 |  | 28 | 14 | 13 | 12 | 17 |  |  |  | 84 |
| **Total** |  |  | **125** | **164** | **110** | **35** | **56** | **80** | **78** | **99** | **106** | **853** |
| High (born 1992-1996) | 1992 | 2008 | 27 | 5 | 14 | 15 | 6 |  |  |  |  | 67 |
| 16 or younger in 2008 | 1993 | 2009 | 8 | 17 | 13 | 18 |  |  |  |  |  | 56 |
|  | 1994 | 2010 | 9 | 12 | 10 |  |  |  |  |  |  | 31 |
|  | 1995 | 2011 | 16 | 11 |  |  |  |  |  |  |  | 27 |
|  | 1996 | 2012 | 10 |  |  |  |  |  |  |  |  | 10 |
| **Total** |  |  | **70** | **45** | **37** | **33** | **6*** | **0** | **0** | **0** | **0** | **185** |

*Not included in Figure 4 due to small group size

**References**

1 Mercer CH, Tanton C, Prah P, Erens B, Sonnenberg P, Clifton S et al. Changes in sexual attitudes and lifestyles in Britain through the life course and over time: findings from the National Surveys of Sexual Attitudes and Lifestyles (Natsal). *Lancet* 2013; 382(9907):1781-1794.
