# Supplementary material for: Chlamydia trachomatis Pgp3 Antibody Population Seroprevalence before and during an Era of Widespread Opportunistic Chlamydia Screening in England (1994-2012)
Source: PLoS One. 2017 Jan 27;12(1):e0152810. doi: 10.1371/journal.pone.0152810 (PMC5271337; doi:10.1371/journal.pone.0152810)
Supplement: S1 Fig — (DOCX) [file pone.0152810.s002.docx]

*Chlamydia trachomatis* Pgp3 antibody population seroprevalence before and during an era of widespread opportunistic chlamydia screening in England (1994-2012)

Supporting Figure 1: Flow chart showing selection of stored sera from Health Survey for England (HSE) participants and inclusion in analyses

16-44 year-old participants in HSE2010/12 & 16-24 year-old female participants in selected years (1994-2012) (n=14,762)

Provided consent for use of stored serum sample

(n=6,882)

Stored sera unavailable or insufficient volume

(n=1,264)

16-44 year-olds in HSE2010 or HSE2012

(n=2,521: 1,402 women; 1,119 men)

16-24 year-old women in selected HSE years (1994-2012)

(n=3,361, 565 seropositive)

Blood sample/consent not provided

(n=7,880)

Pgp3 antibodies detected (seropositive)

(n=546; 372 women, 174 men)

Pgp3 antibodies not detected

(n=1,975)

Study population: stored sera available with sufficient volume for testing

(n=5,618)

**Key**

Included in analysis of age-specific Pgp3 seroprevalence over time

Included in analysis of factors associated with Pgp3 seroprevalence

Included in description of reported chlamydia diagnoses among Pgp3 seropositive

Note: analysis groups are not mutually exclusive, therefore sum of number in each group is greater than total number tested (n=5,618).
