# Supplementary figures and images for: Chlamydia trachomatis Pgp3 Antibody Population Seroprevalence before and during an Era of Widespread Opportunistic Chlamydia Screening in England (1994-2012)
Source: PLoS One. 2017 Jan 27;12(1):e0152810. doi: 10.1371/journal.pone.0152810 (PMC5271337; doi:10.1371/journal.pone.0152810)

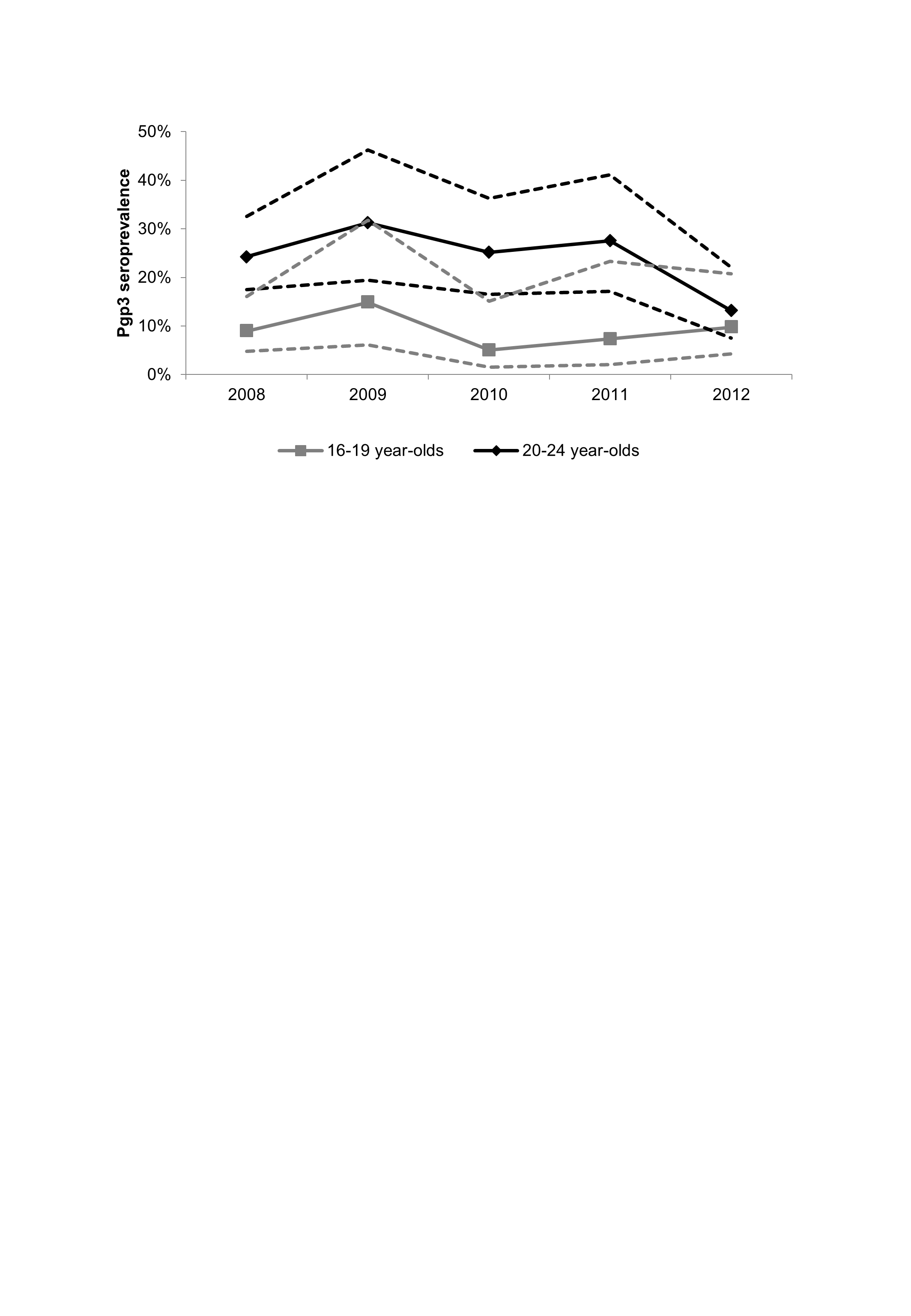

Supplement: S2 Fig — Solid lines show point estimates; dashed lines show 95% confidence intervals. Unweighted denominators: 16–19 year-olds, n = 284; 20–24 year-olds, n = 425. (TIF) [file pone.0152810.s003.tif]
