# Supplementary material for: Chlamydia trachomatis Pgp3 Antibody Population Seroprevalence before and during an Era of Widespread Opportunistic Chlamydia Screening in England (1994-2012)
Source: PLoS One. 2017 Jan 27;12(1):e0152810. doi: 10.1371/journal.pone.0152810 (PMC5271337; doi:10.1371/journal.pone.0152810)
Supplement: S1 Table — (DOCX) [file pone.0152810.s004.docx]

*Chlamydia trachomatis* Pgp3 antibody population seroprevalence before and during an era of widespread opportunistic chlamydia screening in England (1994-2012)

Supporting Table 1: Comparison of reported demographic and behavioural variables between HSE participants and the study population by sex and age group (16-44 year-olds, HSE2010 & HSE2012)

|  | **Women** | | | | | | **Men** | | | | | |
| --- | --- | --- | --- | --- | --- | --- | --- | --- | --- | --- | --- | --- |
|  | **16-24 years** | | | **25-44 years** | | | **16-24 years** | | | **25-44 years** | | |
|  | HSE participants | Study population | Difference | HSE participants | Study population | Difference | HSE participants | Study population | Difference | HSE participants | Study population | Difference |
| **IMD quintile of LSOA area of residence^*^** |  |  |  |  |  |  |  |  |  |  |  |  |
| Least deprived | 18.2% | 16.6% | -1.6% | 18.5% | 17.8% | -0.7% | 18.7% | 17.8% | -0.9% | 17.2% | 16.1% | -1.0% |
| 2 | 18.9% | 19.6% | 0.8% | 18.9% | 19.4% | 0.5% | 18.1% | 19.2% | 1.1% | 19.7% | 19.5% | -0.1% |
| 3 | 21.0% | 21.4% | 0.3% | 21.6% | 21.4% | -0.2% | 21.0% | 19.9% | -1.1% | 21.0% | 21.8% | 0.8% |
| 4 | 21.2% | 21.4% | 0.2% | 19.5% | 19.3% | -0.1% | 18.1% | 17.6% | -0.5% | 21.1% | 21.5% | 0.4% |
| Most deprived | 20.6% | 21.0% | 0.4% | 21.6% | 22.1% | 0.5% | 24.2% | 25.6% | 1.5% | 21.1% | 21.0% | -0.1% |
| **Ethnicity** |  |  |  |  |  |  |  |  |  |  |  |  |
| White | 84.9% | 85.5% | 0.6% | 84.5% | 86.0% | 1.5% | 81.2% | 82.2% | 1.0% | 86.2% | 86.6% | 0.4% |
| Black or Black British | 4.0% | 3.0% | -1.1% | 3.6% | 3.1% | -0.5% | 2.3% | 2.5% | 0.2% | 2.8% | 2.8% | 0.0% |
| Asian or Asian British | 5.7% | 5.3% | -0.4% | 8.5% | 7.8% | -0.7% | 10.4% | 9.7% | -0.6% | 7.7% | 7.6% | -0.1% |
| Mixed | 1.7% | 1.9% | 0.2% | 1.3% | 1.2% | -0.1% | 4.8% | 4.0% | -0.8% | 1.5% | 1.4% | 0.0% |
| Other ethnic groups | 3.8% | 4.4% | 0.5% | 2.0% | 1.9% | -0.1% | 1.3% | 1.5% | 0.2% | 1.8% | 1.6% | -0.2% |
| **Marital status** |  |  |  |  |  |  |  |  |  |  |  |  |
| Single | 78.5% | 77.9% | -0.7% | 18.5% | 18.5% | -0.1% | 86.7% | 86.6% | -0.1% | 22.7% | 22.1% | -0.6% |
| Married | 2.8% | 2.5% | -0.2% | 52.7% | 52.0% | -0.7% | 1.5% | 1.7% | 0.3% | 48.6% | 50.0% | 1.4% |
| Separated | 0.2% | 0.3% | 0.0% | 8.2% | 8.6% | 0.4% | 0.0% | 0.0% | 0.0% | 5.2% | 5.1% | -0.1% |
| Cohabiting | 18.5% | 19.3% | 0.8% | 20.6% | 20.9% | 0.3% | 11.8% | 11.7% | -0.2% | 23.5% | 22.8% | -0.7% |
| **Ever had sex** | 80.4% | 80.2% | -0.2% | 98.4% | 98.5% | 0.0% | 73.7% | 76.5% | 2.8% | 97.3% | 97.2% | -0.1% |
| **Ever diagnosed with chlamydia** | 5.8% | 4.2% | -1.5% | 6.5% | 6.7% | 0.2% | 2.1% | 2.4% | 0.4% | 5.1% | 5.5% | 0.4% |
| **Ever been tested for chlamydia** | 47.6% | 47.7% | 0.1% | 33.5% | 33.2% | -0.4% | 39.1% | 39.9% | 0.9% | 18.0% | 17.7% | -0.4% |

^a^IMD: index of multiple deprivation; LSOA: Lower super output area
